# Comments on Whiley *Legionella* Risk Management and Control in Potable Water Systems: Argument for the Abolishment of Routine Testing. *Int. J. Environ. Res. Public Health* 2017, *14*, 12

**DOI:** 10.3390/ijerph14010102

**Published:** 2017-01-21

**Authors:** Samuel Collins, Jimmy Walker

**Affiliations:** Biosafety, Air and Water Microbiology Group, National Infection Service, Public Health England, Salisbury SP4 0JG, UK; Jimmy.Walker@phe.gov.uk

**Keywords:** *Legionella*, Legionnaires’ disease, routine testing

## Abstract

In their recent article, Whiley makes an interesting case for the abolishment of routine testing in *Legionella* risk management and control plans. Here, we present our views regarding this suggestion, drawing upon our own experiences in the UK. We urge caution against the removal of routine monitoring from guidelines due to the impending public health risks that would result.

We read with great interest the article by Whiley entitled ‘*Legionella* Risk Management and Control in Potable Water Systems: Argument for the Abolishment of Routine Testing’. The author correctly highlights a number of issues that hamper the ability to define the public health risk from water systems contaminated with *Legionella*. Among these issues are the drawbacks associated with the current ISO 11731 ‘gold-standard’ culture method, which include an inability to detect viable but nonculturable (VBNC) cells (the importance of VBNC cells in water systems as well as in infection and disease remains undefined [1]) and high inter- and intra-laboratory variability. We think that there is an argument for improved (cultivation-independent) detection methods that address these issues, enabling accurate enumeration and differentiation of living cells from dead cells from VBNC. As we understand more about the phylogenetic diversity of strains infecting humans [2], perhaps there is an argument for detection strategies targeted to those species, subtypes, or sequence types of *Legionella* known to cause the majority of diseases. Improved detection methods would improve our understanding of the survival of *Legionella* in potable water systems, inform future control measures, and provide more robust data for quantitative microbial risk assessment models.

The author states that routine testing of potable water systems for *Legionella* should be abolished from all guidelines and an emphasis placed on maintaining control measures. However, we advise caution against the wholesale implementation of this advice. We agree that the priority in any potable water system must be to establish and monitor appropriate control regimes highlighted in guidance documents that limit both the potential for *Legionella* to grow and its dissemination. Indeed, risk assessing control strategies forms the basis for many guidance documents worldwide, including the UK Health and Safety Executive Approved Code of Practice L8 [3] and associated technical guidance, the new ASHRAE guidelines [4], and the EWGLI (now ESGLI) technical guidelines [5]. As the author states, an overreliance on routine sampling results (which we appreciate only represent a snapshot in time) can induce a false sense of security [6]. However, so too can an overreliance on control measures alone. To illustrate this point over the past year, we have investigated three potable water systems epidemiologically linked with separate clusters and cases of Legionnaires’ disease (LD). In all systems, control measures (e.g., temperature, and flow) were within recommended parameters and monitored as per national guidelines. However, extensive *Legionella* colonisation was present in key areas, notably where hot and cold water was mixed, e.g., post-thermostatic mixing valves and showers (unpublished data). Colonisation and risk were only identified by epidemiological data, i.e., when cases of LD were reported. Routine testing at these critical control points would have given an early warning and may have prevented these cases from occurring. The availability of planned routine sampling results can also prove very useful in public health investigations of LD, where the colonisation history of a system under investigation (i.e., epidemiologically linked to cases) can help focus investigations and pin-point sources more readily.

The author makes reference to health-care facilities where reducing the exposure of vulnerable individuals is a key priority. Like many potable water systems, a number of hospitals in the UK are colonised with *Legionella*, but not all. It may be a false assumption that *Legionella* are ubiquitous in all potable water systems. For most UK hospitals, a zero-tolerance approach to *Legionella* is based on the assumption that there is no known safe level of *Legionella* and that remedial actions are therefore implemented at the detection of any concentration of *Legionella* [7]. Unlike current Centers for Disease Control and Prevention (CDC) recommendations [8], routine testing plays a crucial role in the water safety plan approach to managing these healthcare systems, proactively identifying risk and offering an additional layer of security to prevent even a single case of LD occurring. This approach, combined with clinical surveillance, has been shown to be effective in identifying and reducing cases of nosocomial LD [9]. It is unlikely that routine testing would be stopped in UK health-care facilities, particularly in systems known to be colonised, as testing provides a means whereby the effectiveness of control regimes can be measured. As Whiley pointed out, there may be an underestimation of the true concentration of *Legionella* in a sample and the variability of the test methods; however, it is logical to assume that the higher the concentration of *Legionella* present in a system the greater the potential for transmission and exposure. An increase in the concentration of *Legionella* should therefore be indicative of a potential problem and be investigated without delay. Moreover, the cost of routine testing and system-wide control measures needs to be carefully balanced against the use of alternative local approaches to protect high risk populations such as point-of-use filters, which, if used permanently, can be costly and may unknowingly introduce infection control risks themselves [10].

Even with the well acknowledged limitations of *Legionella* monitoring, surely the safest approach is to combine risk assessment, control measures (including meticulous monitoring), and routine water testing. Routine testing should be implemented on a system-by-system basis, informed by the risk assessment to reduce inappropriate or meaningless testing and expenditure and take into account the history of the system, the control measures, the previous test results, the population at risk, etc. The absence and detection of low concentrations of *Legionella* by culture (or by other analytical methods) combined with verified control measures are the ultimate reassurances that the microbiological risk has been managed appropriately. After all, cases of LD do not tend to be linked to systems where *Legionella* has not been detected [9,11,12].

Given the controversies and multiple uncertainties in the *Legionella* field, particularly with regard to water system risk assessment and control, there is an urgent need for better and more robust studies to inform future control policies, particularly in light of a global rising incidence of LD and an increasingly older and immunocompromised population.
